# Cerebrospinal fluid-derived circulating tumour DNA better represents the genomic alterations of brain tumours than plasma

**DOI:** 10.1038/ncomms9839

**Published:** 2015-11-10

**Authors:** Leticia De Mattos-Arruda, Regina Mayor, Charlotte K. Y. Ng, Britta Weigelt, Francisco Martínez-Ricarte, Davis Torrejon, Mafalda Oliveira, Alexandra Arias, Carolina Raventos, Jiabin Tang, Elena Guerini-Rocco, Elena Martínez-Sáez, Sergio Lois, Oscar Marín, Xavier de la Cruz, Salvatore Piscuoglio, Russel Towers, Ana Vivancos, Vicente Peg, Santiago Ramon y Cajal, Joan Carles, Jordi Rodon, María González-Cao, Josep Tabernero, Enriqueta Felip, Joan Sahuquillo, Michael F. Berger, Javier Cortes, Jorge S. Reis-Filho, Joan Seoane

**Affiliations:** 1 Vall d'Hebron Institute of Oncology, Vall d'Hebron University Hospital, P. Vall d'Hebron 119-129, 08035 Barcelona, Spain; 2 Department of Pathology, Memorial Sloan Kettering Cancer Center, 1275 York Avenue, New York, New York 10065, USA; 3 Universitat Autònoma de Barcelona, 08193 Barcelona, Spain; 4 Vall d'Hebron Institute of Research, Vall d'Hebron University Hospital, Ps Vall d'Hebron 119-129, 08035 Barcelona, Spain; 5 Center for Molecular Oncology, Memorial Sloan Kettering Cancer Center, 1275 York Avenue, New York, NY 10065, USA; 6 Institució Catalana de Recerca i Estudis Avançats (ICREA), Barcelona, Spain; 7 Department of Surgery, Memorial Sloan Kettering Cancer Center, 1275 York Avenue, New York, NY 10065, USA; 8 Quirón Dexeus University Hospital, 08028 Barcelona, Spain; 9 Human Oncology and Pathogenesis Program, Memorial Sloan Kettering Cancer Center, 1275 York Avenue, New York, NY 10065, USA

## Abstract

Cell-free circulating tumour DNA (ctDNA) in plasma has been shown to be informative of the genomic alterations present in tumours and has been used to monitor tumour progression and response to treatments. However, patients with brain tumours do not present with or present with low amounts of ctDNA in plasma precluding the genomic characterization of brain cancer through plasma ctDNA. Here we show that ctDNA derived from central nervous system tumours is more abundantly present in the cerebrospinal fluid (CSF) than in plasma. Massively parallel sequencing of CSF ctDNA more comprehensively characterizes the genomic alterations of brain tumours than plasma, allowing the identification of actionable brain tumour somatic mutations. We show that CSF ctDNA levels longitudinally fluctuate in time and follow the changes in brain tumour burden providing biomarkers to monitor brain malignancies. Moreover, CSF ctDNA is shown to facilitate and complement the diagnosis of leptomeningeal carcinomatosis.

## Abstract (web summary)

DNA circulating in the plasma of cancer patients carries features of the primary tumour, however such DNA is found in low levels in brain cancer patients. Here, the authors show that circulating tumour DNA can be detected in the cerebral spinal fluid of cancer patients and that this better recapitulates the primary tumour compared to DNA from the plasma.

## Graphical abstract

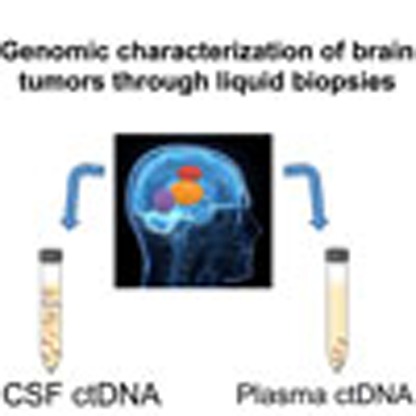

The genomic characterization of tumours is crucial for the optimal diagnosis and treatment of cancer. Given the reported spatial and temporal intratumour heterogeneity, repeated biopsies are required for an adequate characterization of the somatic genetic alterations found in human cancers^1^,^2^. This approach has important limitations, particularly in the case of brain malignancies^3^, due to the restricted and invasive access for sampling tumour material and the challenges to recapitulate the tumour clonal diversity through the analysis of a small fragment of the tumour. Recent work has shown that cell-free circulating tumour DNA (ctDNA) in the plasma could be used to characterize and monitor tumours^4^,^5^,^6^,^7^. ctDNA analysis of patients with brain tumours, however, has revealed either absence or very low levels of tumour DNA in plasma^7^.

The cerebrospinal fluid (CSF) is in intimate contact with tumour cells in central nervous system (CNS) cancers and, recently, ctDNA has been shown to be present in the CSF of patients with brain tumours^8^,^9^. The aim of our work was to determine whether the analysis of CSF ctDNA could be useful for the characterization and monitoring of brain tumours in comparison with plasma ctDNA. We applied hybridization capture-based massively parallel targeted sequencing and/or exome sequencing coupled with droplet digital PCR (ddPCR) to synchronous CSF and plasma-derived ctDNA, and tumour tissue deposits from patients with glioblastoma (GBM), medulloblastoma (Medullo), and brain metastases from lung cancer (BMLC) and from breast cancer (BMBC, six of them subjected to warm autopsies) including breast cancer patients with clinical features suggestive of leptomeningeal carcinomatosis (LC). In this study, we show that ctDNA derived from central nervous system tumours is more abundantly present in the CSF than in plasma. CSF ctDNA can be used to detect brain tumour private mutations and to longitudinally monitor the changes in brain tumour burden. In addition, we provided evidence that the analysis of CSF ctDNA may complement the diagnosis of LC.

## Results

### CSF ctDNA is representative of brain tumours

**Figure 1:**
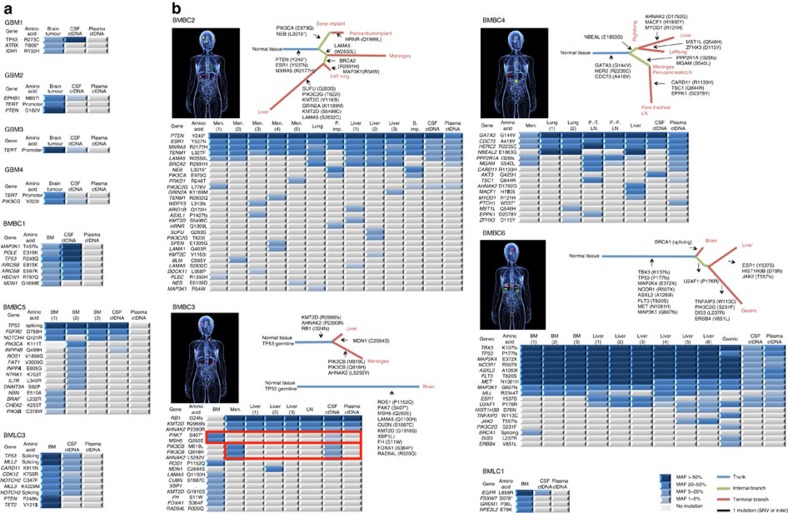
CSF ctDNA better captures the genomic alterations in patients with brain tumours than plasma ctDNA. (**a**,**b**) Analysis of CSF ctDNA, plasma ctDNA and primary brain tumour or metastatic lesions collected simultaneously. Heatmap of the non-silent genetic alterations from each of the twelve cases is shown and phylogenetic trees of the autopsied patients with brain metastasis from breast cancer (BMBC) are represented. Colour key for mutant allelic frequencies (MAFs) is shown. (**a**) Patients with restricted central nervous system (CNS) disease, glioblastoma (GBM), BMBC and brain metastasis from lung cancer (BMLC). (**b**) Patients with CNS and non-CNS disease. BM, brain metastasis; LN, lymph node; Men, meninges; P. Imp, pericardium implant; PT, para-tracheal; PP, peri-pancreatic.

To study and compare the ctDNA present in the CSF with plasma ctDNA, we sequenced DNA obtained from tumour samples, germline DNA (peripheral blood lymphocytes), plasma and CSF of a cohort of 12 patients (4 GBM, 6 BMBCs, 2 BMLCs; Supplementary Table 1). In all cases, except BMBCs, CSF was obtained at the same time than plasma through lumbar puncture or cerebral shunts normally obtaining 1–2 ml of CSF. Tumours and fluids from all six cases of BMBCs were obtained through warm autopsy and the CSF was collected from the cisterna magna. We performed targeted capture massively parallel sequencing and, in all cases, somatic single-nucleotide variants (SNVs), insertion/deletions (indels) and copy-number alterations (CNA) were identified in CSF ctDNA and plasma ctDNA, and validated in the brain tumour tissue from the respective patients (Fig 1a,b, Supplementary Figs 1 and 2, Supplementary Tables 2, 3, Supplementary Data 1, 2, 3). The number of genomic alterations identified through targeted capture sequencing varied from case to case being more abundant in BMBCs and less abundant in GBM cases due to the nature of the genes selected for targeted sequencing. A low rate of mutation capture was observed in the CSF ctDNA from GBM patients indicating that further work is required in order to optimize the detection of ctDNA in GBM cases. CSF ctDNA was identified in all cases while plasma ctDNA was only detected in patients with abundant visceral disease. This is in agreement with previous reports^4^. Our methodology exhibits a detection limit of 2% mutant allelic frequency (MAF)^10^ and patients with low tumour burden present evidence of plasma ctDNA with MAFs below 2% (ref. 4).

In the case of samples from the autopsy material of patients BMBC2, BMBC3, BMBC4 and BMBC6, we had enough number of specimens to infer phylogenetic trees representing the genomic subclonal diversity and be able to identify trunk ubiquitous genetic mutations. Interestingly, trunk mutations were always identified in the CSF ctDNA (Fig. 1b).

In addition, we sequenced the DNA concomitantly extracted from the CSF and plasma in an expansion cohort of 11 patients (2 Medullos, 5 BMLCs, 4 BMBCs) with CNS restricted disease and barely any visceral tumour burden to facilitate the comparison of the contribution of the brain tumour DNA into the CSF or plasma ctDNA. In all cases, CSF ctDNA was detected and harboured gene mutations that were either absent or detected with lower MAFs in plasma ctDNA (Supplementary Fig. 1).

### ctDNA from CSF performs better than plasma

We next sought to determine whether CSF ctDNA would be more representative of the brain lesions than plasma ctDNA. To this end we divided the patients into two groups depending on the amount of extracranial tumour burden (Supplementary Table 4).

**Figure 2:**
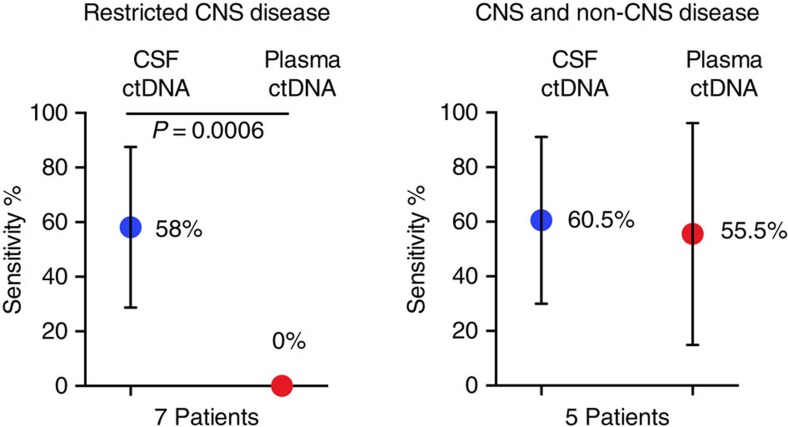
Sensitivity analysis of CSF ctDNA and plasma ctDNA. Sensitivity was inferred based on gene mutations detected in central nervous system (CNS) tumours, which were either identified in CSF or plasma ctDNA (Supplementary Table 5). Data were pooled and the mean with standard deviation error bars is shown. A Mann–Whitney test was used for the analysis and *P* value is shown.

Importantly, in patients with a CNS restricted disease (Fig. 1a, Supplementary Fig. 1), the MAFs in all samples of CSF ctDNA were significantly higher than in plasma (Supplementary Fig. 3) and, moreover, the sensitivity for somatic mutations of the CNS was also significantly higher in CSF ctDNA than plasma ctDNA (Fig. 2, Supplementary Table 5). Some mutations were detected in the CSF or plasma but not in the brain tumour specimen (Fig. 1). These could be potential false positives or mutations not present in the sequenced tumour fragment but present in another region of the brain tumour. In patients with abundant visceral disease (Fig. 1b), the MAFs of the gene mutations in the CSF and plasma ctDNA were comparable (Supplementary Fig. 3).

### CSF ctDNA recapitulates the private mutations from CNS lesions

We have recently observed that, in the context of disseminated disease, brain metastasis might exhibit private gene mutations different from the ones present in the rest of the tumour lesions^11^. We next investigated how CSF and plasma ctDNA might recapitulate the private mutations from CNS lesions in metastatic patients. To answer this question, we analysed the warm autopsy materials of a patient with Li Fraumeni syndrome and a diagnosis of both HER2-positive metastatic breast cancer and esthesioneuroblastoma (BMBC3). Two sets of tumours were present: the breast cancer-derived brain metastasis and, independently, the meningeal implants and liver metastases (Supplementary Fig. 4). The gene mutations of the brain metastasis were not present in the extracranial tumours and, moreover, we identified three private gene mutations (*PIK3CB* M819L, *PIK3CB* Q818H, *AHNAK2* L5292V) exclusively present in the meningeal lesion. The gene mutations with the highest MAFs of the brain metastasis and the private mutations in the meningeal lesions were present in the CSF ctDNA and not in the plasma ctDNA (Fig. 1b, see boxed mutations) indicating that brain private mutations are more represented in the ctDNA from CSF than plasma.

### CSF ctDNA is longitudinally modulated throughout treatments

**Figure 3:**
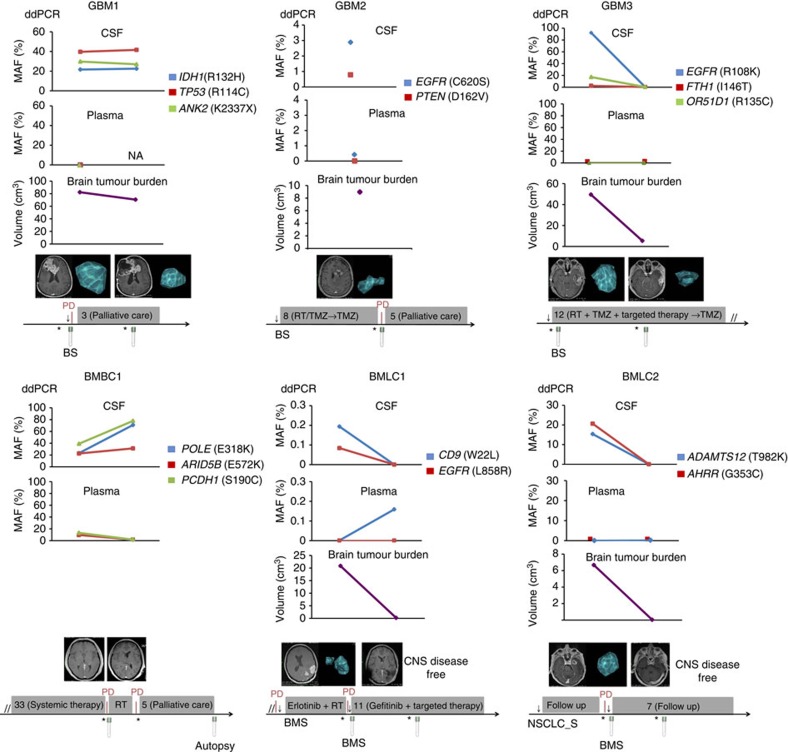
Dynamic changes in CSF ctDNA recapitulate the treatment courses of patients with brain tumours. Longitudinal monitoring of patients with GBM and brain metastases through CSF and plasma ctDNA and the analysis of brain tumour burden. Gene mutations were measured by ddPCR. Tumour volumes were calculated using computer aided planimetric analysis. Timelines reflect the most relevant clinical information for each patient. BS, brain surgery; BMS, brain metastasis surgery; CNS, central nervous system; NSCLC_S, non-small cell lung cancer surgery; PD, progressive disease; RT, radiotherapy; TMZ, temozolomide. Asterisk and arrow indicate time of magnetic resonance imaging and surgical procedure, respectively. Grey boxes indicate therapy or follow up, and their duration is provided in months.

To address whether the amount of ctDNA present in the CSF could fluctuate with time and be representative of the brain tumour progression, we obtained concomitantly CSF and plasma from six patients (GBM and metastatic breast and lung cancer patients with brain metastasis) at sequential time points (Supplementary Table 1, Fig. 3). In all cases, there was a minimal or absent extracranial disease. Brain lesions were identified using magnetic resonance imaging and brain tumour burden was quantified using computer aided planimetric analysis (Supplementary Table 6). The tumour somatic genomic alterations, previously identified in the tumours by exome sequencing, were determined in the CSF-derived DNA of the patients through ddPCR (Fig. 3). As expected, the MAFs in all samples of CSF ctDNA were higher than in plasma (Supplementary Table 7). Importantly, MAFs of CSF ctDNA decreased with surgical resection and/or responses to systemic therapy and increased with tumour progression (Fig. 3). The MAFs were modulated over time and followed the same trend as the variation in brain tumour burden. These results indicated that CSF may be a useful biomarker to monitor tumour progression and response to treatment.

### CSF ctDNA complements the diagnosis of LC

The identification of CSF ctDNA led us to the hypothesis that cell-free DNA in the CSF could be used as a diagnostic tool for LC. The diagnosis of LC relies on the detection of malignant cells in the CSF of patients with clinical symptoms. Diagnosis of LC is not trivial and its misdiagnosis has important clinical implications. To define whether the analysis of CSF ctDNA can be employed to enhance the sensitivity of the detection of LC by cytopathologic analysis of CSF, we performed standard of care cytopathologic analysis and CSF ctDNA sequencing in the same samples obtained from three breast cancer patients with clinical signs and symptoms suggestive of LC.

**Figure 4:**
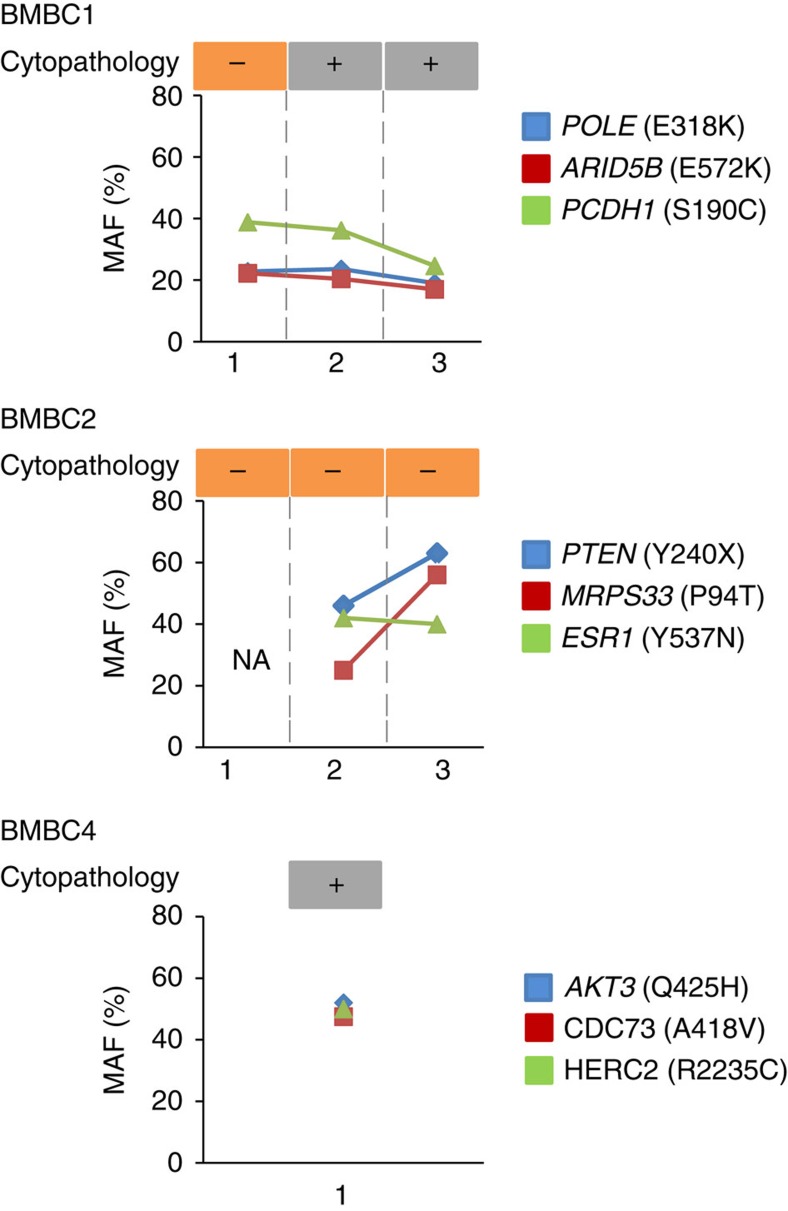
Analysis of CSF ctDNA as a diagnostic tool for leptomeningeal carcinomatosis in three metastatic breast cancer patients. The results of serial clinical cytopathology analyses are shown in the upper part of the graph. In the lower part, mutant allelic frequencies (MAFs) measured by ddPCR in the same CSF samples are depicted. NA, not available.

Importantly, there were discrepancies between the cytology and our CSF ctDNA analysis (Fig. 4). In BMBC2, although three cytopathologic analyses yielded negative results, we detected ctDNA with MAFs ranging from 20 to 50% in the two CSF samples that were available (Fig. 4). Given that LC was confirmed at the autopsy of BMBC2, our results indicated that the CSF ctDNA analysis detected disease at a level not detectable by cytopathologic analysis. In BMBC1, one of the cytopathologic analysis was discordant with the presence of CSF ctDNA, while in BMBC4 the results of the cytopathologic analysis and the CSF ctDNA were in agreement. In both cases, BMBC1 and BMBC4, LC was confirmed at the autopsy. In summary, our results build a proof-of-concept that opens the possibility to use CSF ctDNA to complement the diagnosis of LC. Of note, in the case of patients with brain metastasis and clinical signs suggestive of LC, the analysis of CSF ctDNA can be misleading since it will be difficult to discern whether the ctDNA in the CSF is originated from the LC or the brain metastasis. Further studies will be needed to consolidate this methodology for LC diagnosis.

## Discussion

In this study, we identified and characterized ctDNA in the CSF of patients with brain lesions and compared it with plasma ctDNA. We showed that CSF ctDNA is more representative of brain tumour genomic alterations than plasma and putative actionable gene mutations and CNA (that is, *EGFR*, *PTEN*, *ESR1, IDH1, ERBB2, FGFR2*) can be identified. We observed that CSF ctDNA has a significantly higher sensitivity than plasma for CNS genomic alterations and can be used to detect brain tumour private mutations and to monitor brain tumour progression. In addition, we provided evidence that the analysis of CSF ctDNA may complement the diagnosis of LC.

One of the hallmarks of GBM is the fact that all tumours relapse. Once diagnosed, the GBM tumour is surgically resected and then the patient receives radio- and chemotherapy treatments. Even when the surgical resection is complete, the tumour invariably relapses. Importantly, the relapsed tumour tends to evolve under treatment and present different genomic alterations than the primary tumour^12^. Surgical procedures (resection and biopsies) are seldom indicated in relapsed GBM limiting its genomic characterization and precluding the treatment of the relapsed GBM based on genomic information. CSF ctDNA provides a minimally invasive method to assess the genomic alterations of the relapsed tumour helping to select the optimal treatment dictated by the molecular characteristics of the brain cancer.

On the other hand, patients with brain metastasis exhibit a dismal prognosis and are usually recalcitrant to treatments. It is known that, most likely due to the special environment of the brain, the genomic alterations of brain metastasis differ from the ones of the visceral malignancies and primary tumours^11^,^12^,^13^,^14^,^15^. The identification of the brain metastasis-specific genomic alterations through CSF ctDNA might facilitate the design of tailored treatments to target brain metastasis hopefully increasing the clinical response of these deadly lesions.

In a context where the oncology field expects that therapeutic approaches will be dictated and guided by the genomic features of tumours, the presence of CSF ctDNA will be fundamental to the correct molecular diagnosis and treatment of brain tumours. Altogether, our results indicate that CSF ctDNA can be exploited as a ‘liquid biopsy' of brain tumours opening a novel avenue of research in CNS circulating biomarkers with an important impact in the future characterization, diagnosis, prognosis and clinical managing of brain cancer.

## Methods

### Patients

Breast cancer patients with brain metastasis were enrolled as part of the Vall d'Hebron Institute of Oncology (VHIO) Warm Autopsy Program. Patients with breast cancer and lung cancer with brain metastasis, and GBM and medulloblastoma were enrolled as part of VHIO Prospective Translational Program, which studies plasma and CSF-derived biomarkers. Patients with lung cancer with brain metastasis were enrolled as part a collaborative effort with Dexeus University Hospital (Barcelona, Spain) and the research was approved by the local institutional review board (IRB)/ethics committee of both hospitals. VHIO Warm Autopsy Program and the Prospective Translational Program were approved by the IRB of Vall d'Hebron University Hospital (Barcelona, Spain). Informed consent was obtained from all patients.

### DNA extraction

The diagnosis of each metastatic lesion was confirmed on review of routine hematoxylin and eosin-stained slides^6^. Ten 8-μm thick sections from representative fresh frozen metastasis biopsies/resections were cut, stained with nuclear fast red and microdissected with a needle under a stereomicroscope to ensure >80% of tumour cell content, as previously described^16^. DNA from microdissected tumour samples was extracted using DNeasy Blood and Tissue Kit (Qiagen, USA), and germline DNA from peripheral blood lymphocytes (‘buffy coat') was extracted using the QIAamp DNA Mini Kit (Qiagen) according to manufacturer's instructions. CSF-derived and plasma-derived circulating cell-free DNA was extracted with the QIAamp Circulating Nucleic Acid Kit (Qiagen, Valencia, CA, USA), as previously described^6^. DNA was quantified using the Qubit Fluorometer (Invitrogen).

### Targeted capture massively parallel sequencing

DNA samples from CNS tumours (primary brain tumours or CNS metastases) of 23 cases, non-CNS metastases, CSF and plasma samples as well as germline DNA were subjected to targeted capture massively parallel sequencing at the Memorial Sloan Kettering Cancer Center Integrated Genomics Operation (iGO), using the Integrated Mutation Profiling of Actionable Cancer Targets (MSK-IMPACT) platform^17^ targeting all exons of 341 cancer genes harbouring actionable mutations. For four additional cases with breast cancer and brain metastases (BMBC1-4) were analysed with a customized breast cancer panel, targeting all exons of 254 genes recurrently mutated in breast cancer and/or related to DNA repair (Supplementary Data 1) was also performed. For these four cases, of the 595 genes captured, 107 genes were common to both targeted capture platforms (that is, 488 unique genes), and were employed for validation. By applying the methods described above to each targeted capture platform independently, the validation rate of somatic mutations (SNVs and indels) affecting the exons of 107 genes present in both platforms was >96% (Supplementary Table 3).

Targeted sequencing was performed as previously described^6^,^17^,^18^. In brief, 20–450ng of DNA was used to prepare barcoded sequence libraries (New England Biolabs, Kapa Biosystems), which were pooled at equimolar concentrations for hybridization exon capture (Nimblegen SeqCap).

Paired-end 100-bp reads were generated on the Illumina HiSeq2000 (San Diego, CA), and reads were aligned to the reference human genome hg19 using the Burrows-Wheeler Aligner^19^. Local realignment, duplicate removal and base quality recalibration were performed using the Genome Analysis Toolkit^20^. Somatic SNVs were called using MuTect^21^, and small insertions and deletions (indels) were called using Strelka^22^, VarScan 2 (ref. 23) and SomaticIndelDetector^17^. All candidate mutations were reviewed manually using the Integrative Genomics Viewer^24^. Somatic mutations with allelic fractions of <1% and/or supported by <2 reads were disregarded. The mean sequence coverage of each target exon was subjected to a loess normalization to adjust for bias in nucleotide composition (G+C) and compared with the diploid normal sample. Gene copy-number profiles were generated using circular binary segmentation^17^.

### Exome sequencing of tumour DNA and normal DNA

DNA (500ng) extracted from brain tumour and germline samples from GBM1, GBM2 and GBM3, BMBC1, BMLC1 and BMLC2 cases were subjected to exome sequencing. An average of 100 million 100-bp paired-end reads were generated for each sample, equivalent to an average depth of 260 × (range of 190–315 × ). Exome sequencing was performed using the Nextera Rapid Capture Exome kit (37 Mb; Illumina) on an Illumina HiSeq 2000 instrument using a validated protocol^25^ and according to the manufacturer's recommendations (Macrogen).

### ddPCR and quantification of circulating tumour-specific DNA

ddPCR of plasma and CSF were performed using the QX200 Droplet Digital PCR system (Bio-Rad) according to manufacturer's protocols and the literature^26^. TaqMan-based quantitative PCR assays were designed to specifically detect point mutations and corresponding wild-type alleles as selected by exome sequencing of primary brain tumours or brain metastases. Primer sequences are provided in Supplementary Table 8. Ten nanograms of genomic DNA extracted from tumour tissue and germline DNA from peripheral blood lymphocytes was used for digital PCR analysis. In some cases, lower amounts of DNA (for example, 1–5 ng) were used, due to CSF and plasma DNA yield limitations.

### The phylogenetic tree generation

Phylogenetic trees were constructed using the maximum parsimony method. The trunks of the trees were rooted by a germline DNA sequence that did not have any of the somatic mutations. Trunk, branch and sub-branches lengths are proportional to the number of mutations.

### Statistical analysis

A Mann–Whitney test was performed for statistical analysis. Data in graphs are presented as means±s.d.

## Additional information

**Accession codes**: Whole-exome and targeted capture massively parallel sequencing have been deposited in the Sequence Read Archive (SRA) under accession code SRP049647.

**How to cite this article:** De Mattos-Arruda, L. *et al.* Cerebrospinal fluid-derived circulating tumour DNA better represents the genomic alterations of brain tumours than plasma. *Nat. Commun.* 6:8839 doi: 10.1038/ncomms9839 (2015).

## Supplementary Material

Supplementary Information Supplementary Figures 1-4 and Supplementary Tables 1-8

Supplementary Data 1 List of the customized breast cancer and MSK-IMPACT platforms (254 breast cancer-associated genes and 341 cancer-associated genes) involved in the targeted capture massively parallel sequencing employed in this study. The lists of 488 unique cancer-associated genes derived from both platforms and genes in common to both platforms are also provided.

Supplementary Data 2 Somatic single nucleotide variants (SNVs) and insertions and deletions (indels) from the IMPACT platform present in patients with glioblastoma, brain metastasis from lung cancer (BMLC) and brain metastasis from breast cancer (BMBC) subjected to warm autopsies. SNVs and indels from the customized breast cancer present in four BMBC (cases 1-4) subjected to warm autopsies.

Supplementary Data 3 Sequencing metrics for the MSK-IMPACT and breast panels.

## References

[b1] Gerlinger M. *et al.* Intratumor heterogeneity and branched evolution revealed by multiregion sequencing. N. Engl. J. Med. 366, 883–892 (2012). 22397650 10.1056/NEJMoa1113205 PMC4878653

[b2] Sottoriva A. *et al.* Intratumor heterogeneity in human glioblastoma reflects cancer evolutionary dynamics. Proc. Natl Acad. Sci. USA 110, 4009–4014 (2013). 23412337 10.1073/pnas.1219747110 PMC3593922

[b3] Omuro A. & DeAngelis L. M. Glioblastoma and other malignant gliomas: a clinical review. JAMA 310, 1842–1850 (2013). 24193082 10.1001/jama.2013.280319

[b4] Murtaza M. *et al.* Non-invasive analysis of acquired resistance to cancer therapy by sequencing of plasma DNA. Nature 497, 108–112 (2013). 23563269 10.1038/nature12065

[b5] Dawson S. J. *et al.* Analysis of circulating tumor DNA to monitor metastatic breast cancer. N. Engl. J. Med. 368, 1199–1209 (2013). 23484797 10.1056/NEJMoa1213261

[b6] De Mattos-Arruda L. *et al.* Capturing intra-tumor genetic heterogeneity by de novo mutation profiling of circulating cell-free tumor DNA: a proof-of-principle. Ann. Oncol. 25, 1729–1735 (2014). 25009010 10.1093/annonc/mdu239 PMC6276937

[b7] Bettegowda C. *et al.* Detection of circulating tumor DNA in early- and late-stage human malignancies. Sci. Transl. Med. 6, 224ra24 (2014). 10.1126/scitranslmed.3007094 PMC4017867 24553385

[b8] Segal M. B. Extracellular and cerebrospinal fluids. J. Inherit. Metab. Dis. 16, 617–638 (1993). 8412010 10.1007/BF00711896

[b9] Pan W., Gu W., Nagpal S., Gephart M. H. & Quake S. R. Brain tumor mutations detected in cerebral spinal fluid. Clin. Chem. 61, 514–522 (2015). 25605683 10.1373/clinchem.2014.235457 PMC5412506

[b10] Cheng D. T. *et al.* Memorial sloan kettering-integrated mutation profiling of actionable cancer targets (MSK-IMPACT): a hybridization capture-based next-generation sequencing clinical assay for solid tumor molecular oncology. J. Mol. Diagn. 17, 251–264 (2015). 25801821 10.1016/j.jmoldx.2014.12.006 PMC5808190

[b11] Brastianos P. K. *et al.* Genomic characterization of brain metastasis reveals branched evolution and potential therapeutic targets. Cancer Discov. 5, 1–14 (2015). 10.1158/2159-8290.CD-15-0369 PMC4916970 26410082

[b12] Johnson B. E. *et al.* Mutational analysis reveals the origin and therapy-driven evolution of recurrent glioma. Science 343, 189–193 (2014). 24336570 10.1126/science.1239947 PMC3998672

[b13] Ding L. *et al.* Genome remodelling in a basal-like breast cancer metastasis and xenograft. Nature 464, 999–1005 (2010). 20393555 10.1038/nature08989 PMC2872544

[b14] Saunus J. M. *et al.* Integrated genomic and transcriptomic analysis of human brain metastases identifies alterations of potential clinical significance. J. Pathol. 237, 363–378 (2015). 26172396 10.1002/path.4583

[b15] Paik P. K. *et al.* Next-generation sequencing of stage IV squamous cell lung cancers reveals an association of PI3K aberrations and evidence of clonal heterogeneity in patients with brain metastases. Cancer Discov. 5, 610–621 (2015). 25929848 10.1158/2159-8290.CD-14-1129 PMC4643059

[b16] Hernandez L. *et al.* Genomic and mutational profiling of ductal carcinomas in situ and matched adjacent invasive breast cancers reveals intra-tumour genetic heterogeneity and clonal selection. J. Pathol. 227, 42–52 (2012). 22252965 10.1002/path.3990 PMC4975517

[b17] Won H. H., Scott S. N., Brannon A. R., Shah R. H. & Berger M. F. Detecting somatic genetic alterations in tumor specimens by exon capture and massively parallel sequencing. J. Vis. Exp. 80, e50710 (2013). 10.3791/50710 PMC3947962 24192750

[b18] Natrajan R. *et al.* Characterization of the genomic features and expressed fusion genes in micropapillary carcinomas of the breast. J. Pathol. 232, 553–565 (2014). 24395524 10.1002/path.4325 PMC4013428

[b19] Li H. & Durbin R. Fast and accurate short read alignment with Burrows-Wheeler transform. Bioinformatics 25, 1754–1760 (2009). 19451168 10.1093/bioinformatics/btp324 PMC2705234

[b20] DePristo M. A. *et al.* A framework for variation discovery and genotyping using next-generation DNA sequencing data. Nat. Genet. 43, 491–498 (2011). 21478889 10.1038/ng.806 PMC3083463

[b21] Cibulskis K. *et al.* Sensitive detection of somatic point mutations in impure and heterogeneous cancer samples. Nat. Biotechnol. 31, 213–219 (2013). 23396013 10.1038/nbt.2514 PMC3833702

[b22] Saunders C. T. *et al.* Strelka: accurate somatic small-variant calling from sequenced tumor-normal sample pairs. Bioinformatics 28, 1811–1817 (2012). 22581179 10.1093/bioinformatics/bts271

[b23] Koboldt D. C. *et al.* VarScan 2: somatic mutation and copy number alteration discovery in cancer by exome sequencing. Genome Res. 22, 568–576 (2012). 22300766 10.1101/gr.129684.111 PMC3290792

[b24] Robinson J. T. *et al.* Integrative genomics viewer. Nat. Biotechnol. 29, 24–26 (2011). 21221095 10.1038/nbt.1754 PMC3346182

[b25] Lamble S. *et al.* Improved workflows for high throughput library preparation using the transposome-based Nextera system. BMC Biotechnol. 13, 104 (2013). 24256843 10.1186/1472-6750-13-104 PMC4222894

[b26] Forshew T. *et al.* Noninvasive identification and monitoring of cancer mutations by targeted deep sequencing of plasma DNA. Sci. Transl. Med. 4, 136ra68 (2012). 10.1126/scitranslmed.3003726 22649089

