# Supplementary material for: Cerebrospinal fluid-derived circulating tumour DNA better represents the genomic alterations of brain tumours than plasma
Source: Nat Commun. 2015 Nov 10;6:8839. doi: 10.1038/ncomms9839 (PMC5426516; doi:10.1038/ncomms9839)
Supplement: Supplementary Information — Supplementary Figures 1-4 and Supplementary Tables 1-8 [file ncomms9839-s1.pdf]

Supplementary Information

Supplementary Figure 1

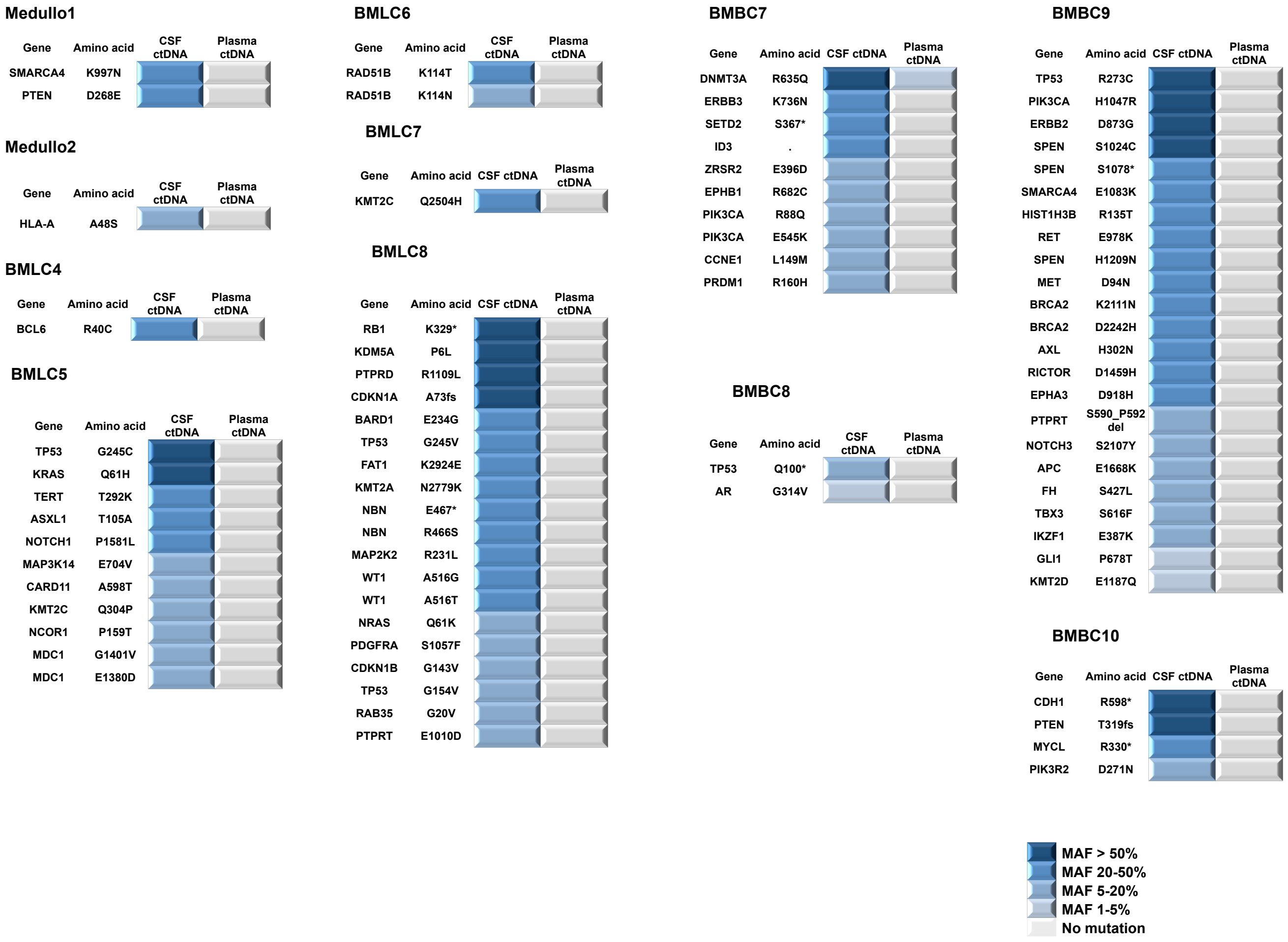

Supplementary Figure 1. Expansion cohort comparing CSF ctDNA and plasma ctDNA collected simultaneously in patients with brain tumors. Heatmap of the non-silent genetic alterations from each of the cases is shown. Color key for mutant allelic frequencies (MAFs) is represented.

Supplementary Figure 2

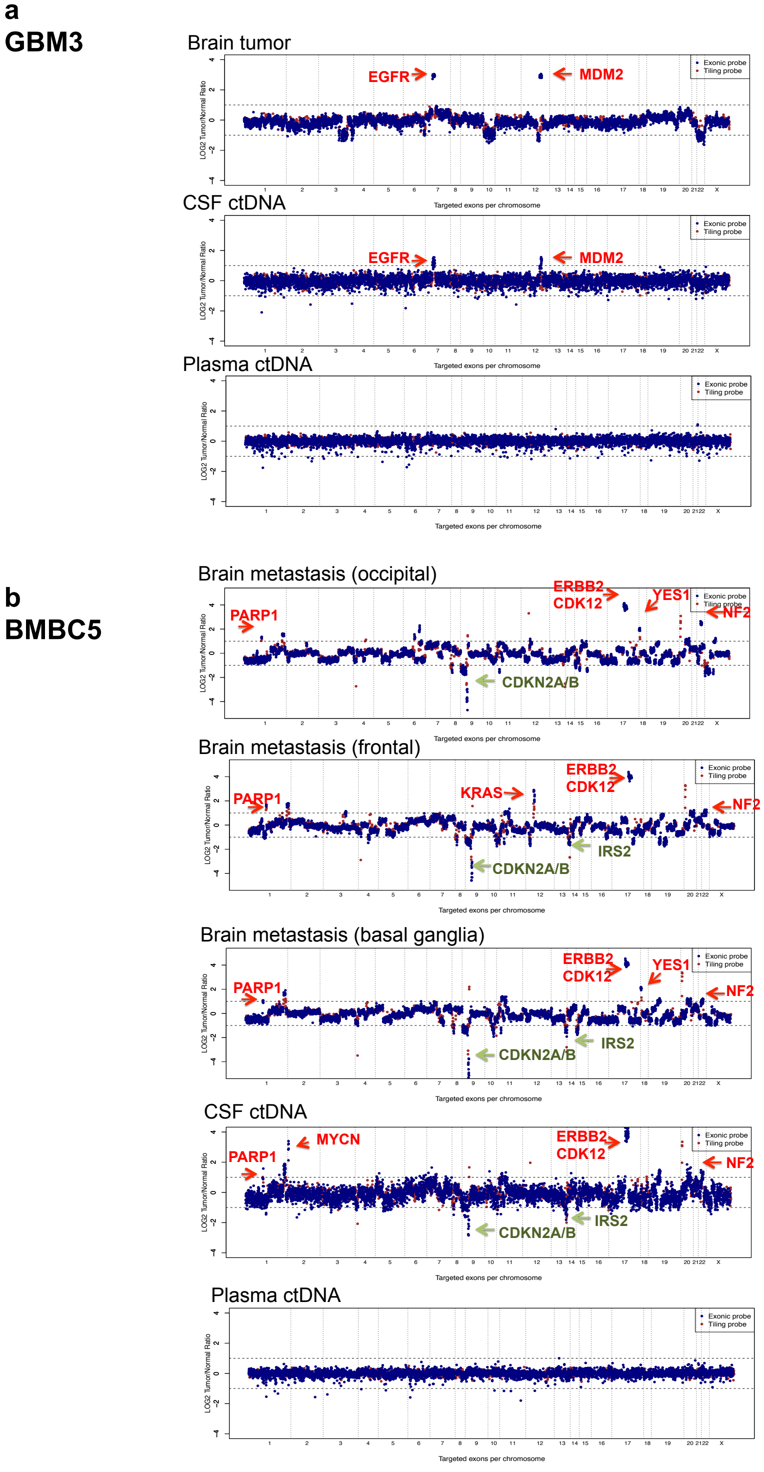

Supplementary Figure 2. Gene copy number alterations across the brain tumor, CSF, plasma which were collected simultaneously in **(a)** a patient with glioblastoma **(b)** a patient with brain metastasis from breast cancer (BMBC). Note that plasma ctDNA did

not play a role in such patients with minimal or absent CNS disease.

Supplementary Figure 3

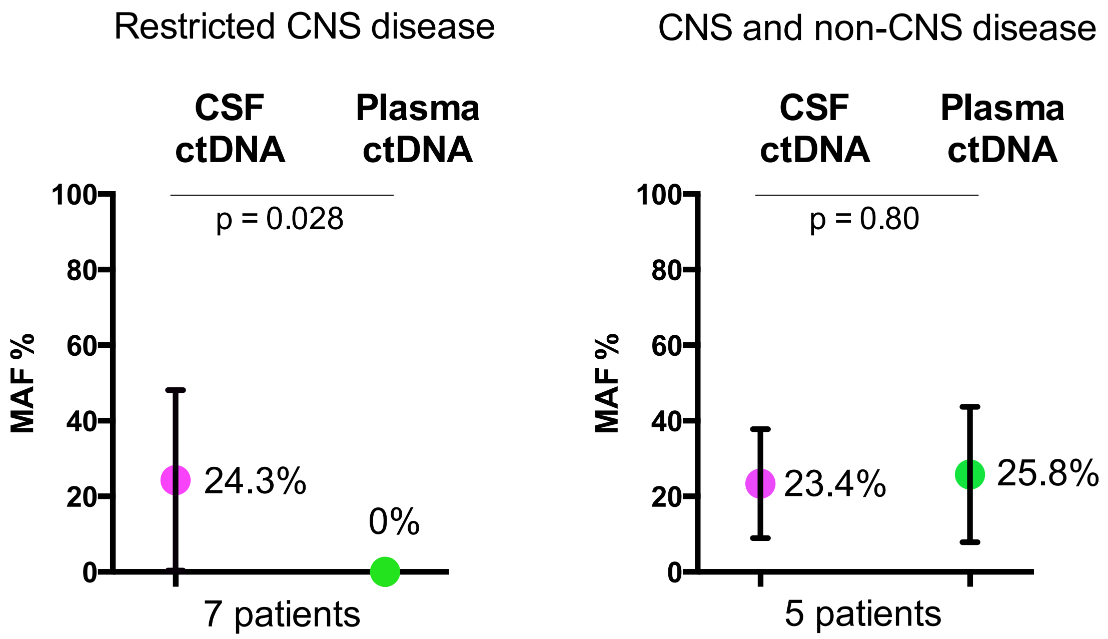

Supplementary Figure 3. Comparison of the mutant allelic frequencies (MAF) of CSF ctDNA and plasma ctDNA in CNS restricted disease and CNS and non-CNS disease. Data were pooled and the mean with SD error bars is shown (Mann-Whitney test).

Supplementary Figure 4

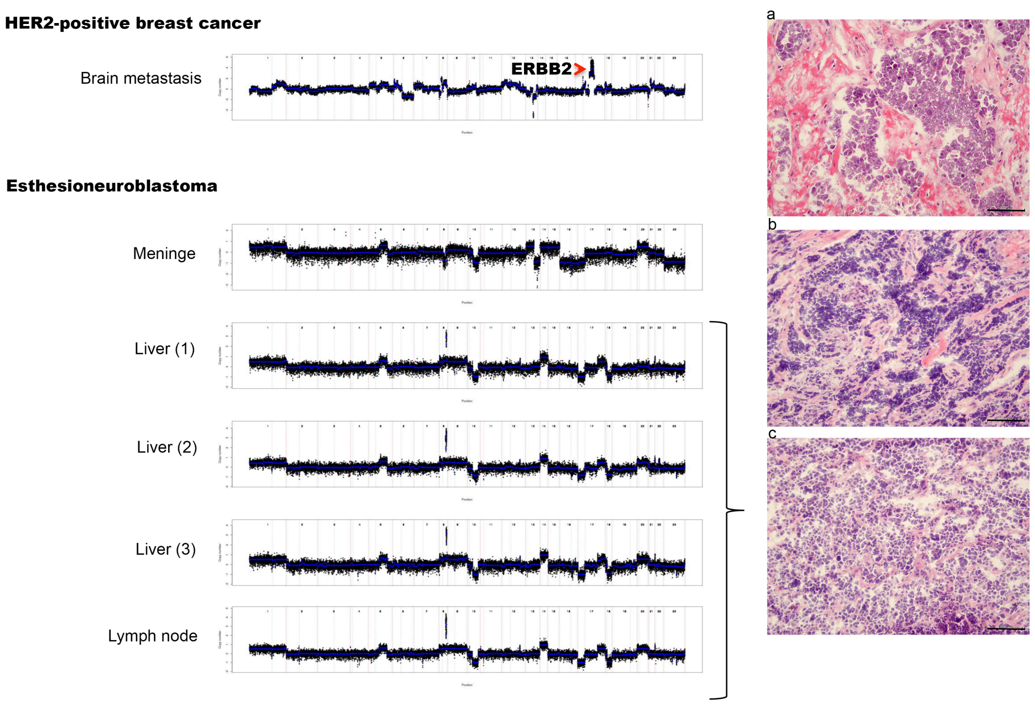

Supplementary Figure 4. Genomic analysis of a patient with Li Fraumeni syndrome and a diagnosis of both HER2-positive metastatic breast cancer and esthesioneuroblastoma (BMBC3). The brain metastasis was inferred to be originated from the HER2-positive metastatic breast cancer, as *ERBB2* gene copy amplification is shown at the genomic position 17q12. Micrographs representing **(a)** brain metastasis; **(b)** meninges; **(c)** liver metastasis. Scale bar = 500 μm

Supplementary Table 1. Patients' clinical details.

Case	Tumor type	Biopsy site	Clinical summary
GBM1	Glioblastoma	Brain tumor (secondary GBM)	33 year-old woman with diffuse low-grade astrocytoma in the right frontal and temporal lobes. The patient was operated and received chemotherapy and WBRT. After 5 years free of disease, she developed neurologic symptoms and had documented recurrence on MRI. Following treatment with temozolomide, she progressed later on and she was subjected to partial resection of the brain tumor, which showed evidence of secondary glioblastoma, IDH1 positive. Patient had fast clinical deterioration and received palliative care until she passed away.
GBM2	Glioblastoma	Primary brain tumor	52 year-old man with diagnosis of glioblastoma. Following partial resection of the brain tumor, patient received WBRT plus concomitant and adjuvant temozolomide. He progressed months later as observed on MRI. After clinical deterioration, he received palliative care until he passed away.
GBM3	Glioblastoma	Primary brain tumor	64 year-old woman with diagnosis of temporal glioblastoma (IDH1 negative, *EGFR* mutant). She was subjected to partial resection of the brain tumor. Then, she was treated with a targeted therapy plus temozolomide, concomitantly with WBRT.
GBM4	Glioblastoma	Primary brain tumor (secondary GBM)	54 year-old man with anaplastic astrocytoma. The tumor was subjected to suboptimal exeresis and the patient received chemotherapy and WBRT. After 12 months, a new exeresis was performed which showed evidence of secondary glioblastoma. Patient received a new line of chemotherapy, and upon progression, was enrolled in a clinical trial. Patient had no clinical benefit and passed away.
BMBC1	Metastatic breast cancer	Brain metastasis	56 year-old woman diagnosed with ductal invasive ER-positive, HER2-negative early breast cancer (T2N0M0).

			After 10 years, she developed bone metastasis. She received multiple lines of treatments with stable disease for 33 months when she presented neurologic symptoms. Following MRI and serial cytological spinal fluid examination, a diagnosis of leptomeningeal carcinomatosis was established and WBRT was administered. Further disease progression in the brain was observed and the patient was enrolled in a palliative care program due to poor performance status.
BMBC2	Metastatic breast cancer	Meningeal implants	35 year-old woman diagnosed with ductal invasive ER-positive, HER2-negative breast cancer (T1cN0M0) in 2007. She had breast conservative surgery and received standard adjuvant therapy. In 2010, a biopsy confirmed bone metastasis and the patient received endocrine-based therapy and local RT. After multiple lines of endocrine, chemotherapy and targeted therapy due to bone and lung progression, the patient developed neurologic symptoms. Signs of leptomeningeal carcinomatosis were detected on the MRI but three serial spinal fluid cytological analyses were negative. After WBRT and a new line of chemotherapy, visceral disease progression was observed.
BMBC3	Metastatic breast cancer	Brain metastasis and meningeal implants	33 year-old woman diagnosed with ductal invasive carcinoma ER-positive, HER2-positive breast cancer (cT2N2M1) in 2006. The patient received first-line therapy with the anti-HER2 trastuzumab and chemotherapy, achieving almost complete response. After bone progression, she received multiple lines of trastuzumab combined with endocrine or chemotherapy. Subsequently, a diagnosis of Li-Fraumeni syndrome (germline *TP53* mutation) was confirmed. In 2009, she presents disease progression in CNS, bone and breast received WBRT and was treated with targeted therapies. In 2012, due to a right nasal cavity mass and palpable cervical lymph node, a diagnosis of a secondary primary tumor

			(esthesioneuroblastoma) was made and the patient received local RT combined with cisplatin and etoposide. Further disease progression was observed in brain, bone (a biopsy was done and had HER2-positive status) and visceral sites and the patient received further lines with cytotoxic agent and anti-HER2 therapy.
BMBC4	Metastatic breast cancer	Meningeal implants	43 year-old woman diagnosed with locally advanced ductal invasive ER-positive, HER2-positive breast cancer (cT4bN2M0) in 2006. After neoadjuvant chemotherapy, patient was submitted to mastectomy, adjuvant endocrine treatment, and trastuzumab. In 2009, she developed bone and liver metastases and received several lines of anti-HER2-based systemic therapies, including a targeted therapy and trastuzumab. After presenting mentonian paresthesias, leptomeningeal carcinomatosis was clinically diagnosed and treated with WBRT. Two months after the patient passed away.
BMBC5	Metastatic breast cancer	Brain metastasis	36 year-old woman diagnosed with invasive ductal carcinoma ER-negative, HER2-positive breast cancer (cT3N3M1) in 2012. The patient received first-line therapy with the anti-HER2 trastuzumab plus paclitaxel; following successive visceral and brain progression (2013), patient received WBRT and more 3 lines of anti-HER2 therapies combined with cytotoxic and targeted agents until she passed away in 2014.
BMBC6	Metastatic breast cancer	Brain metastasis	37 year-old woman diagnosed with invasive ductal carcinoma ER-positive (pT2N1M0) in 1993. She had a mastectomy and received standard adjuvant therapy. In 1995, bone metastasis was diagnosed and the patient received endocrine-based therapy and local RT. In 2013, after multiple lines of endocrine, chemotherapy and targeted therapy due to bone and hepatic progression, the patient developed neurologic symptoms. A brain MRI confirmed the diagnosis of brain metastases and WBRT

			was administered. Patient received a new line of chemotherapy and passed away.
BMLC1	Non-small cell lung cancer	Brain metastasis	36 year-old woman with non-small cell lung adenocarcinoma (stage IV, *EGFR* mutation). Patient was subjected to partial resection of thoracic disease (lobectomy and pleural implants) and received post-operative chemotherapy. Following new surgical intervention, received another post-operative chemotherapy. After disease progression, she received treatment with erlotinib. After two years, she developed neurologic symptoms and had documented progressive disease documented in brain MRI. She was subjected to incomplete resection of the left parietal-occipital brain metastasis, which harbored an activating *EGFR* mutation (exon 21) and carried on treatment with erlotinib. Following brain RT, and due to persistence of brain disease on MRI, patient was operated again. After two months, patient had evidence of progressive disease in mediastinum and was treated with targeted therapies. Patient had partial response in mediastinum and no evidence of recurrence in the brain.
BMLC2	Non-small cell lung cancer	Brain metastasis	69 year-old man with non-small cell lung adenocarcinoma. Patient was operated (pT2N2M0, *EGFR* and *ALK* negative) and kept in clinic-radiological follow up. Four months after, he developed neurologic symptoms and had documented progressive disease on brain MRI. He was subjected to complete resection of the left temporal brain metastasis, and further received WBRT, being disease free.
BMLC3	Non-small cell lung cancer	Brain metastasis	65 year-old man with non-small cell lung adenocarcinoma (T4N3M0). The patient is treated with chemotherapy and radiotherapy, achieving complete response. Twelve months after, he developed neurologic symptoms and had documented progressive disease on brain MRI. He was subjected to complete resection of the cerebellar

			brain metastasis, being disease free.

Abbreviations: ALK, anaplastic lymphoma kinase; CNS, central nervous system; EGFR, epidermal growth factor receptor; ER, estrogen receptor, GBM, glioblastoma; MRI, magnetic resonance imaging; WBRT, whole brain radiotherapy.

Supplementary Table 2. Expansion cohort of patients in which CSF ctDNA and plasma ctDNA are compared.

Patient ID	Tumor Type
**Brain metastasis from breast cancer**	
**BMBC7**	Brain metastasis from ER-positive / HER2-negative breast cancer
**BMBC8**	Brain metastasis from ER-negative / HER2-negative breast cancer
**BMBC9**	Brain metastasis from ER-positive / HER2-negative breast cancer
**BMBC10**	Brain metastasis from ER-positive / HER2-negative breast cancer
**Brain metastasis from lung cancer**	
**BMLC4**	Non-small cell lung cancer (large cell carcinoma)
**BMLC5**	Non-small cell lung cancer (adenocarcinoma, *KRAS* mutation)
**BMLC6**	Non-small cell lung cancer (large cell carcinoma)
**BMLC7**	Non-small cell lung adenocarcinoma (*ALK* translocation)
**BMLC8**	Non-small cell lung adenocarcinoma
**Primary brain tumors**	
**Medullo1**	Medulloblastoma (classic type)
**Medullo2**	Medulloblastoma (anaplastic type)

Supplementary Table 3. Validation rate of somatic mutations (SNVs and indels) affecting the exons of 107 genes present in both IMPACT and Breast Cancer platforms.

Case	Validation rate of IMPACT (using breast panel as the validation set)	Validation rate of breast panel (using IMPACT as the validation set)
**BMBC1**	90.9% (20/22)	100% (20/20)
**BMBC2**	97.4% (37/38)	100% (37/37)
**BMBC3**	100% (21/21)	100% (21/21)
**BMBC4**	91.7% (11/12)	100% (11/11)
**Overall**	96.7 % (89/92)	100% (89/89)

Supplementary Table 4. Tumor burden of patients with central nervous system disease (CNS)-restricted disease and CNS and non-CNS (disseminated disease).

Case	Site of metastases	Lesions on last imaging scans
**CNS restricted**		
**GBM1**	CNS	Lesion in the right frontal lobe (55x60mm)
**GBM2**	CNS	Lesion in the corpus callosum (39x16x19 mm).
**GBM3**	CNS	Lesion in the left temporal lobe (50x28 mm).
**GBM4**	CNS	Right parieto-occipital mass (85x58x53 mm).
**BMBC1**	CNS	Right thalamic mass (5.6 x 4.2mm), meningeal implants (not measurable).
	Non-CNS	Mild pleural, pericardial implants and bone metastasis (not measurable).
**BMBC5**	CNS	Multiple supra and infratentorial brain metastases: left frontal (15x19 mm), right parietal (13x24mm), basal ganglia (12x10 mm), occipital (10x8 mm).
	Non-CNS	Hepatic subcentimeter lesion in segment IV.
**BMLC3**	CNS	Pre-surgical cerebellar brain metastasis (37x33x34 mm).
	Non-CNS	No evidence of thoracic disease.

CNS and non-CNS (disseminated disease)		
BMBC2	CNS	Meningeal implants in left and right parietal convexity.
	Non-CNS	Liver with about 60-70% of the parenchyma involved with metastasis (greater lesions: 43x40mm and 27x27mm, liver longer axis 27cm), pleural and abdominal effusion and multiple bone metastases (not measurable).
BMBC3	CNS	Left temporo-occipital (30x16mm), right frontal (7x6 mm), extra-parenchymatous falx cerebri (10x11mm), esphenoidal lesion (not measurable).
	Non- CNS	Moderate left pleural effusion, hepatic subcentimeter lesion in segment II, multiple bone metastases (not measurable).
BMBC4	CNS	Meningeal implants in brain and cervical spinal cord (not measurable).
	Non-CNS	Liver (21x18mm, 15x21mm, 39x15mm, 29x22mm, and others - longer axis 21cm, 30-40% parenchyma involved), para-tracheal lymph node (8x7mm), peri-pancreatic lymph node (13x10mm), multiple bone metastases (not measurable).
BMBC6	CNS	Pre and post central gyrus lesions (18x18 mm), multiple left fronto-parietal gyrus lesions, posterior fossa (vermis lesion 3 mm).
	Non-CNS	Multiple liver metastases: segment V/VIII (69x55 mm), segment IV (59x43 mm), segment III (18x17mm). Peritoneal implants and multiple bone metastases (not measurable).
BMLC1	CNS	Left parieto-temporal brain lesion (32x23x26 mm) and left parieto-occipital lesion (27x14 mm).
	Non-CNS	Pulmonary lesion in left upper lobe (5.8x4 mm).

Supplementary Table 5. Analysis of sensitivity for central nervous system (CNS) and non-CNS disease, CSF ctDNA and plasma ctDNA.

CNS restricted					
	**MSK-IMPACT**	**MSK-IMPACT and breast panel**	**N of all SNVs and indels**	**Present in CSF and CNS**	**Present in plasma and CNS**
**GBM1**	Yes		5	1/3 (33.3%)	0/3 (0%)
**GBM2**	Yes		3	1/3 (33.3%)	0/3 (0%)
**GBM3**	Yes		1	1/1 (100%)	0/1 (0%)
**GBM4**	Yes		4	1/2 (50%)	0/2 (0%)
**BMBC1**		Yes	21	6/7 (85.7%)	0/7 (0%)
**BMBC5**	Yes		15	4/15 (26.6%)	0/15 (0%)
**BMLC3**	Yes		9	7/9 (77.8%)	0/9 (0%)
**Mean**			8.4	58%	0%
**CNS and non-CNS (disseminated disease)**					
**BMBC2**		Yes	28	6/16 (37.5%)	6/16 (37.5%)
**BMBC3**		Yes	17	6/16 (100%)	3/16 (50%)
**BMBC4**		Yes	16	3/5 (60%)	5/5 (100%)
**BMBC6**	Yes		18	8/10 (80%)	9/10 (90%)
**BMLC1**	Yes		4	1/4 (25%)	0/4 (0%)
**Mean**			16.6	60.5%	55.5%

Supplementary Table 6. Tumor volume measurements as per computer aided planimetric analyses.

Patient ID	Volume of brain lesions (cm^3)	
	Time Point 1	Time Point 2
**GBM1**	82.24	70.32
**GBM2**	N/A	8.98
**GBM3**	49.36	4.7
**BMLC1**	20.99	0
**BMLC2**	6.71	0

Abbreviation: N/A, not available.

Supplementary Table 7. Gene mutations and their respective mutant allelic frequencies in the CSF ctDNA and plasma ctDNA as determined per digital PCR.

Case	Gene	Amino acid Change	CSF 1	CSF 2	Plasma 1	Plasma 2
**GBM1**	IDH1	R132H	21.8%	22.7%	0.0%	N/A
	TP53	R114C	40.0%	42.0%	0.0%	N/A
	ANK2	K2337X	30.0%	27.2%	0.0%	N/A
**GBM2**	EGFR	C620S	2.9%	N/A	0.4%	N/A
	PTEN	D162V	0.8%	N/A	0.0%	N/A
**GBM3**	EGFR	R108K	92.6%	0.0%	0.3%	0.0%
	FTH1	I146T	2.0%	0.0%	0.0%	0.0%
	OR51D1	R135C	17.0%	0.0%	0.0%	0.0%
**BMBC1**	POLE	E318K	22.8%	70.9%	10.7%	1.0%
	ARID5B	E572K	22.2%	30.9%	9.8%	2.0%
	PCDH1	S190C	38.8%	77.9%	13.6%	2.0%
**BMLC1**	CD9	W22L	0.2%	0.0%	0.0%	0.2%
	EGFR	L858R	0.1%	0.0%	0.0%	0.0%
**BMLC2**	ADAMTS12	T982K	15.5%	0.0%	0.0%	0.2%
	AHRR	G353C	20.8%	0.0%	0.0%	0.0%
**Median (range)**	-	-	20.8% (0.1-92.6)	0% (0-77.9)	0% (0-13.6)	0.1% (0-2.0)

Abbreviation: N/A, not available.

Supplementary Table 8. List of primers and probes used for ddPCR

Gene	Aminoacid change	Forward primer	Reverse primer	Variant probe (FAM)	Reference probe (VIC)
IDH1	R132H	CTTGTGAGTGGATGGGTAAAACCTA	CCAACATGACTTACTTGATCCCCATA	ATCATAGGTCATCATGC	CATCATAGGTCGTCATGC
EGFR	C620S	ACGCCGGCCATGTGT	ACTTTCCACTCACCCGTAGGT	CACCTGAGCCATCC	CCACCTGTGCCATCC
EGFR	R108K	CAGTGGAGCGAATTCCTTTGGA	ACTGCTAAGGCATAGGAATTTTCGT	CAGATCATCAAAGGAAAT	CAGATCATCAGAGGAAAT
POLE	E318K	CTCATCACCAACAGGGAGATTGT	CGGGTTCATTGAAGACACAAAAGG	CAGAAGATATTAAAGATTTT	CAGAAGATATTGAAGATTTT
PTEN	Y240X	ACACGACGGGAAGACAAGTT	TGATATCACCACACACAGGTAACG	AGGGAACTCAAACTACATG	AGGGAACTCAAAGTACATG
AHRR	G353C	CCCTCTAAACCCCAACAGGAA	GGCCAGCGTCAGTCTGTT	AGAGAGAGCTGCGTTTT	AGAGAGCGGCGTTTT
CDC73	A418V	AGAAGAAAAGACCAGATGCAACCA	GGGCTGGTCTACTACTCTATAAGGT	AACACTAATTACAGTGCCC	CACTAATTGCAGTGCCC
CD9	W22L	CAAGTGCATCAAATACCTGCTGTTC	ACAGGTGGGCCCTGAGA	CTTCATCTTCTTGGTGAGTG	CTTCATCTTCTGGGTGAGTG
ADAMTS12	T982K	TGCCAAGAACCATGATGAACCTT	GCTGGAGGCCACACAGA	TTGGGTTTCCTTTTCACATC	TTGGGTTTCCTTGTCACATC
HERC2	R2235C	GTTATGCACGATGAGTTTGGAGAAG	CACGGTGATTTTGCCCTTTGG	CACTGTGACTTGCATCA	CACTGTGACTCGCATCA
FTH1	I146T	GTGACTTCATTGAGACACATTACCTGAA	GCTCCCATCTTGCGCAAGT	CAATTCTTTGGTGGCTTT	CCAATTCTTTGATGGCTTT
PTEN	D162V	CACAAGAGGCCCTAGATTTCTATGG	CAGATCCAGGAAGAGGAAAGGAAAA	ACCAGAGTCAAAAAG	ACCAGAGACAAAAAG
ANK2	K2337X	CAGGCAGCTGTAGTGTAGCA	GCTGCCTCCTCAGTCAGT	AGGTGTCTCTTAAGCTAA	TGTAGGTGTCTCTTTAGCTAA
ESR1	Y537N	CTGTACAGCATGAAGTGCAAGAAC	TGGGCGTCCAGCATCTC	TGCCCCTCAATGAC	TGGTGCCCCTCTATGAC
TP53	R114C	CTACTGGGACGGAACAGCTT	CTGTGCGCCGGTCTCT	CACAAACACACACCTCA	CACAAACACGCACCTCA
MRPS33	P94T	ACGACTAAAGAAGCTTCGTGGAAAG	TTGAGGGACCAACACTATTTCCTTTT	CCTTTCTTTGTTTTCTC	CCTTTCTTTGGTTTCTC
EGFR	L858R	GCAGCATGTCAAGATCACAGATT	CCTCCTTCTGCATGGTATTCTTTCT	AGTTTGGCCCGCCCAA	AGTTTGGCCAGCCCAA
OR51D1	R135C	CCTGCTGGCCATGGCT	CGCAATGGGTGGCAAATGG	CCACAAAGCAGTCAAA	CCACAAAGCGGTCAAA
ARID5B	E572K	TCCTACGTCCTGAAGCAAGAAATTC	AGCTAGGCATGTGGGAATGG	AGGATAAACTCTTAAAGAAAA	AAGGATAAACTCTTAGAGAAAA
PCDH1	S190C	TCACCCGAGACTGGAGAGATC	CTCATAGCTCTCCCGCTGTTC	CGATCCAGACATGTCT	CGATCCAGAGATGTCT
AKT3	Q425H	TCTTTCTTTTTACAGCTTGTACCTCCTTT	TGTAATAGTCTGAGCTGTAAATTCTT CATCAAAA	CAGATGTTACGTGAGGTT	TCTCAGATGTTACTTGAGGTT
